# The impact of COVID-19 vaccination on psychiatric symptoms in schizophrenia patients: A retrospective study

**DOI:** 10.1097/MD.0000000000034016

**Published:** 2023-06-09

**Authors:** Xinghu Wu, Yu Gao, Xiaoling Cheng, Xiaoyong Lin

**Affiliations:** a Department of Psychiatry, Hainan Province Anning Hospital, Haikou, China; b Quanzhou Third Hospital, Fujian, China.

**Keywords:** COVID-19, psychiatry, schizophrenia, vaccination

## Abstract

The objective was to investigate the impact of COVID-19 vaccination on anxiety, depression, stress perception, and psychiatric symptoms in patients with schizophrenia, and to explore severity of psychiatric symptoms is associated with vaccine hesitancy in individuals with schizophrenia. Mental health symptoms were evaluated in 273 hospitalized schizophrenia patients who received COVID-19 vaccination, and in 80 patients who refused vaccination, both before and after immunization. The study assessed the effects of vaccination on psychiatric symptoms and the potential association between vaccination behavior and psychological distress. Our findings suggest that COVID-19 vaccination is associated with a small worsening of schizophrenia symptoms in older inpatients. Moreover, vaccination behavior may increase anxiety, depression, and stress perception in hospitalized schizophrenia patients, which has implications for the mental health care team working in the context of the pandemic. The study highlights the importance of monitoring the mental health status of patients with schizophrenia during the COVID-19 pandemic, particularly in relation to vaccination behavior. Further research is needed to better understand the mechanisms underlying the observed effects of COVID-19 vaccination on psychiatric symptoms in patients with schizophrenia.

## 1. Introduction

Since 2019, Corona Virus Disease 2019 (COVID-19) has rapidly spread worldwide and has become a significant global public health crisis. It is widely acknowledged that vaccination is critical for increasing population immunity among vulnerable individuals.^[1]^ Severe mental illnesses, such as schizophrenia, have been identified as the second-highest risk factor for COVID-19 mortality and linked to an increased risk of new coronavirus infection,^[1]^ moreover, COVID-19 patients are vulnerable to stressful situations and that the prevalence of depressive and anxiety symptoms is increased.^[2–4]^ Hospitalized individuals with schizophrenia are particularly at risk of COVID-19 infection and transmission.^[5–7]^ Hospitalized individuals with schizophrenia are particularly at risk of COVID-19 infection and transmission.^[8]^ However, it is generally recognized that people suffering from schizophrenia are unlikely to make decisions about vaccines. This reluctance to vaccinate may increase the risk of COVID-19 infection among individuals with schizophrenia.^[9,10]^

Despite the increased risk of severe COVID-19 in people with schizophrenia, little is known about the impact of COVID-19 vaccination on mental symptoms, and there is limited data on how psychiatric symptoms in schizophrenia are related to COVID-19 vaccine uptake. This study aims to investigate the effect of COVID-19 vaccination on mental symptoms in hospitalized patients with schizophrenia, as well as the association between the severity of psychiatric symptoms and vaccine hesitancy, and contribute to the understanding of the interplay between COVID-19 vaccination, mental symptoms, and vaccine hesitancy in hospitalized patients with schizophrenia. This knowledge could inform mental health care teams and public health strategies to provide appropriate support and interventions for individuals with schizophrenia during the ongoing COVID-19 pandemic.

## 2. Methods

### 2.1. Recruitment

The study collected information from 368 inpatients with schizophrenia between February 21, 2021, and June 28, 2022, through online and offline surveys. Clinical information was collected anonymously, and researchers did not have access to information that could identify individual participants during collection. A qualified physician supervised the questionnaire filling process, and authors had access to identifiable information during or after data collection. All subjects had a clinical diagnosis of schizophrenia based on ICD-10,^[11]^and patients with co-occurring personality disorders, mental impairment, or substance abuse disorders were excluded. Of the 368 participants, 285 received the COVID-19 vaccine, and 83 declined. During the study period, 12 patients in the vaccination group and 3 patients in the non-vaccination group were released and not included in the analysis. Mental symptoms of hospitalized patients were assessed by a competent psychiatrist twice: once at baseline and once a month after vaccination, using the Patient Health Questionnaire-9 (PHQ-9), Generalized Anxiety Disorder-7 (GAD-7), Perceived Stress Scale (PSS), and Positive and Negative Syndrome Scale (PANSS).

### 2.2. Questionnaire

#### 2.2.1. GAD-7.

The GAD-7 is a 7-item generalized anxiety inventory. Each entry was scored 4, 3 for virtually daily, 2 for more than 1 week, 1 for days away, and 0 for not at all. The final score is the sum of the 7 items’ grades, with a total score range of 0 to 21 points.^[12]^

#### 2.2.2. PHQ-9.

There are a total of 9 items in the PHQ-9, and please pick the response that best reflects the circumstance based on genuine feelings during the previous 2 weeks, making a judgment based on the initial impression. The following are some notes about options: There was no or very little time (during the last week, no more than 1 day when such scenarios arose); Days: proportion of time (the disease existed for 1 to 3 days in the previous week); More than half of the time: a significant amount of time (within the past week, there have been such cases on about 4 days). Almost every day: the most of the time (within the past week, there have been 5 to 7 days).^[13]^

#### 2.2.3. PSS.

PSS assesses how much stress people believe they are under as a result of an experience that may cause emotional or bodily reactions. The grading method of a 5-point scale was used, and assessment indicators comprised the participants’ attitudes, replies, and recognition. Reversal questions are 4, 5, 6, 7, 9, 10, and thirteen. Its standard “1” signifies never, “2” denotes almost never, “3” represents occasionally, “4” implies frequently, and “5” denotes always.^[14]^

Cronbach’s alpha values for PH9, GAD-7, PSS, and PANSS were 0.832, 0.768, and 0.822.0.818, respectively.^[2]^

### 2.3. Statistical analysis

The Kolmogorov–Smirnov test was used with the statistical analysis program SPSS23.0 to assess if the normality hypothesis was satisfied for each dependent variable. To compare the 2 groups, the *t* test was used, and the measurement data that followed the normal distribution were represented as (mean ± SD). The chi-square test was used to compare the 2 groups, and the count results were given as a rate. Statistics were considered significant when *P* = .05. The relationship between PANSS and various indicators was explored by multiple factor linear regression and Pearson correlation analysis.

## 3. Results

The demographic and clinical characteristics of the study participants are presented in Table 1. There were no significant differences between the COVID-19 vaccination group and the control group in terms of baseline age, gender, or duration of illness. The use of sedatives/hypnotics was slightly higher in the COVID-19 group, but there were no significant differences in other medication use. No allergic reactions were reported in the COVID-19 vaccination group during the observation period. None of the patients in either group were infected with the COVID-19 virus during the study.

**Table 1:** Epidemiology and general clinical information.

	Unvaccinated patients (n = 80)	Vaccinated patients (n = 273)	*P* value
Age	55.3 (9.5)	58.6 (8.9)	>.05
Gender (male/female)	152 (55.6)/121 (44.3)	46 (57.5)/34 (42.5)	>.05
Course of disease medications	26.3 (8.6)	28.6 (9.2)	>.05
Antipsychotics (%)	94.1%	92.5%	>.05
Antidepressants (%)	4.3%	5.0%	>.05
Sedatives/hypnotics (%)	53.1%	61.3%	**<.01**
Mood stabilizers (%)	12.8%	13.7%	>.05
Symptoms post vaccination			
Fever	0.4%	0%	–
Cough	6.5%	5.0%	>.05
Headache	0%	1.2%	–
Dizziness	0%	0%	–

These values are expressed as the mean ± SD for age and course of disease, and as number (%) for gender.

The comparison of groups revealed that the baseline levels of psychiatric symptoms in the 2 groups were comparable (*P* > .05). PHQ-9, GAD-7, and PSS measures were used to assess the 2 groups’ perceptions of depression, anxiety, and stress. The findings were comparable to mental symptoms, with no significant difference between the 2 groups. (Table 2).

**Table 2:** Baseline mental status of the 2 groups of patients.

	Unvaccinated patients (n = 80)	Vaccinated patients (n = 273)	*P* value
Baseline PANSS score	64.8 (8.7)	66.8 (9.8)	>.05
Positive subscale score	16.5 (4.3)	17.1 (4.5)	>.05
Negative subscale score	18.7 (4.1)	19.3 (5.2)	>.05
General psychopathology subscale score	27.9 (5.4)	29.2 (4.8)	>.05
Baseline PHQ-9	5.3 (1.5)	4.9 (1.3)	>.05
Baseline GAD-7	4.5 (1.4)	4.2 (1.2)	>.05
Baseline PSS	17.8 (2.5)	18.1 (2.2)	>.05

GAD-7 = Generalized Anxiety Disorder Scale-7, PHQ-9 = Patient Health Questionnaire-9, PSS = Perceived Stress Scale.

Following that, we looked into whether COVID-19 vaccine might alter the mental symptoms of hospitalized patients. The PANSS total score, negative subscale, and general psychopathology subscale scores all rose substantially following immunization, as indicated in Table 3 (overall score: *P* < .01; negative: *P* < .01; general: *P* < .01). Furthermore, following vaccination, levels of depression, anxiety, and stress perception rose to varied degrees (PHQ-9: *P* < .05; GAD-7: *P* < .05; PSS: *P* < .01). However, no statistically significant changes were seen in the unvaccinated group.

**Table 3:** Mental state of 2 groups before and after vaccination.

	Unvaccinated (Before/Post)	*P* value	Vaccinated (Before/Post)	*P* value
Total PANSS score	64.8 (8.7)/65.6 (9.7)	>.05	66.8 (9.8)/71.9 (11.8)	**<.01**
Positive subscale score	16.5 (4.3)/16.3 (4.8)	>.05	17.1 (4.5)/18.2 (5.3)	>.05
Negative subscale score	18.7 (4.1)/19.3 (6.5)	>.05	19.3 (5.2)/22.1 (7.2)	**<.05**
General psychopathology subscale score	27.9 (5.4)/28.5 (5.8)	>.05	29.2 (4.8)/33.4 (7.6)	**<.05**
PHQ-9	5.3 (1.5)/5.5 (1.4)	>.05	4.9 (1.3)/7.8 (1.6)	**<.05**
GAD-7	4.5 (1.4)/4.7 (1.3)	>.05	4.2 (1.2)/6.9 (1.5)	**<.05**
PSS	17.8 (2.5)/18.3 (2.5)	>.05	18.1 (2.2)/26.3 (3.5)	**<.01**

GAD-7 = Generalized Anxiety Disorder Scale-7, PHQ-9 = Patient Health Questionnaire-9, PSS = Perceived Stress Scale.

We attempted to utilize multiple linear regression to construct a model to investigate the factors influencing the severity of mental symptoms following COVID-19 immunization but were ultimately unable in obtaining a satisfactory model. According to early findings, age and stress perception may be associated factors (Table 4).

**Table 4:** Regression analysis between psychiatric symptoms and variables.

	Beta value		*P* value
	Lower 95% CI	Upper 95% CI	
Age	0.105	0.319	**<.01**
Gender	−3.747	0.601	>.05
Course of disease	−0.051	0.195	>.05
PHQ-9	−1.276	0.148	>.05
GAD-7	−0.444	0.920	>.05
PSS	0.498	1.144	**<.05**

Multiple linear regression.

GAD-7 = Generalized Anxiety Disorder Scale-7, PHQ-9 = Patient Health Questionnaire-9, PSS = Perceived Stress Scale.

**Significant correlation at .01 level (bilateral).

After that, we performed Pearson correlation analysis to determine the link between psychiatric symptoms and numerous variables. Our findings corroborated the preceding conclusions. The severity of mental symptoms was positively correlated with age and stress perception (*P* < .01) (Table 5).

**Table 5:** Correlation analysis of psychotic symptoms and variables.

	Age	Gender	Course of disease	PHQ-9	GAD-7	PSS
Age	1	0.063	−0.015	−0.031	−0.006	0.091
Gender	0.063	1	0.083	−0.080	−0.003	−0.014
Course of disease	−0.015	0.083	1	0.019	0.034	−0.043
PHQ-9	−0.031	0.080	0.019	1	−0.073	−0.106
GAD-7	−0.006	−0.003	0.034	−0.073	1	0.041
PSS	−0.091	−0.014	−0.043	−0.106	0.041	1
PANSS	**0.242** **	−0.058	0.042	−0.121	0.058	**0.313** **

Pearson correlation analysis.

GAD-7 = Generalized Anxiety Disorder Scale-7, PHQ-9 = Patient Health Questionnaire-9, PSS = Perceived Stress Scale.

** Significant correlation at 0.01 level (bilateral).

## 4. Discussion

Our study suggests that COVID-19 vaccination may lead to a slight worsening of mental symptoms in hospitalized schizophrenia patients, especially in elderly patients and those who perceive external pressure highly. The increase in PANSS scores is likely due to vaccination behavior rather than the vaccine itself. First and foremost, before vaccination, all participants were informed of any potential side effects. Expected adverse effects may cause an anti-placebo reaction, exacerbating preexisting psychological problems. Second, many participants in this study were long-term hospitalized mental patients with isolated social networks who were unaware of societal events such as the pandemic. Vaccination behavior may be an external source of stress, reminding people of pandemic knowledge and triggering anxiety or depression.

The COVID-19 pandemic has had profound societal consequences, impacting not only economic and political policies, but also the physical and mental health of individuals.^[15,16]^ The COVID-19 vaccine has been effective in controlling the spread of the virus and has helped us overcome the pandemic.^[17]^ However, the potential risks and side effects of the vaccine may cause anxiety or fear among certain individuals, especially vulnerable groups with severe mental illness.^[18–21]^ In this study, we investigated the relationship between COVID-19 vaccination and psychiatric symptoms in hospitalized patients with schizophrenia. Our findings indicate that the severity of psychiatric symptoms is associated with age and perceived stress. Additionally, we found that COVID-19 vaccination may slightly increase PANSS scores as well as anxiety, depression, and perceived stress in patients with schizophrenia.

Previous studies have shown that individuals with mental illnesses are at a higher risk of contracting COVID-19,^[22,23]^ which has led to a focus on the preventive impact of vaccination.^[24,25]^ However, it is important to acknowledge that individuals with mental illnesses have a different experience of the world due to poor judgment, limited perception, low education level, and anxiety or fear. The vaccination behavior and potential adverse effects can cause psychological stress and affect their mental state, which are all linked to the severity of mental symptoms.

Overall, our findings suggest that healthcare providers should be aware of the potential impact of vaccination on mental health, especially in vulnerable groups with severe mental illness. It is crucial to provide adequate information and support to these individuals before and after vaccination to reduce psychological stress and improve their overall well-being.

## 5. Limitations

However, while our study provides valuable insights into the impact of COVID-19 vaccination on mental symptoms in patients with schizophrenia, it is important to acknowledge the limitations of the study, including the sample size and potential selection bias. The strengths lie in the longitudinal design, focus on a vulnerable population, comprehensive assessment, and implications for mental health care. Further research is warranted to explore the underlying mechanisms and to confirm and expand upon our findings in larger and more diverse populations.

## 6. Conclusion

According to our study findings, COVID-19 vaccination behavior may increase anxiety, depression, stress perception, and psychiatric symptoms in patients with schizophrenia, especially in older patients. Therefore, in order to provide the best care for individuals with schizophrenia, psychiatric personnel should fully understand the potential benefits and drawbacks of COVID-19 immunization. Additionally, psychiatrists should closely monitor the mental health of older patients after COVID-19 immunization and take appropriate measures to reduce psychological stress if necessary. This will help to minimize the negative impact of vaccination on the mental health of vulnerable patients with schizophrenia. Our study highlights the importance of considering mental health factors when implementing vaccination strategies in individuals with schizophrenia.

## Author contributions

**Conceptualization:** Xinghu Wu, Yu Gao, Xiaoyong Lin.

**Data curation:** Xinghu Wu, Yu Gao.

**Formal analysis:** Yu Gao.

**Investigation:** Xinghu Wu, Xiaoling Cheng.

**Methodology:** Yu Gao, Xiaoling Cheng.

**Resources:** Xiaoyong Lin.

**Software:** Yu Gao, Xiaoyong Lin.

**Supervision:** Xiaoyong Lin.

**Validation:** Xiaoling Cheng, Xiaoyong Lin.

**Writing – original draft:** Xinghu Wu.

**Writing – review & editing:** Xiaoling Cheng, Xiaoyong Lin.
